# Comparison of total immunoglobulin G antibody responses to different protein fragments of *Plasmodium vivax* Reticulocyte binding protein 2b

**DOI:** 10.1186/s12936-022-04085-x

**Published:** 2022-03-04

**Authors:** Caitlin Bourke, Eizo Takashima, Li-Jin Chan, Melanie H. Dietrich, Ramin Mazhari, Michael White, Jetsumon Sattabongkot, Wai-Hong Tham, Takafumi Tsuboi, Ivo Mueller, Rhea Longley

**Affiliations:** 1 The Walter and Eliza Hall Institute of Medical Research, 3052 Parkville, Australia; 2 Department of Medical Biology, The University of Melbourne, 3052 Parkville, Australia; 3 Division of Malaria Research, Proteo-Science Center, Ehime University, Matsuyama, Japan; 4 Infectious Disease Epidemiology and Analytics G5 Unit, Institut Pasteur, Paris, France; 5 Mahidol Vivax Research Unit, Faculty of Tropical Medicine, Mahidol University, Bangkok, Thailand

**Keywords:** *Plasmodium vivax*, Reticulocyte binding protein 2b, IgG, Serological markers

## Abstract

**Background:**

*Plasmodium vivax* is emerging as the dominant and prevalent species causing malaria in near-elimination settings outside of Africa. Hypnozoites, the dormant liver stage parasite of *P. vivax*, are undetectable to any currently available diagnostic test, yet are a major reservoir for transmission. Advances have been made to harness the naturally acquired immune response to identify recent exposure to *P. vivax* blood-stage parasites and, therefore, infer the presence of hypnozoites. This in-development diagnostic is currently able to detect infections within the last 9-months with 80% sensitivity and 80% specificity. Further work is required to optimize protein expression and protein constructs used for antibody detection.

**Methods:**

The antibody response against the top performing predictor of recent infection, *P. vivax* reticulocyte binding protein 2b (PvRBP2b), was tested against multiple fragments of different sizes and from different expression systems. The IgG induced against the recombinant PvRBP2b fragments in *P. vivax* infected individuals was measured at the time of infection and in a year-long observational cohort; both conducted in Thailand.

**Results:**

The antibody responses to some but not all different sized fragments of PvRBP2b protein are highly correlated with each other, significantly higher 1-week post-*P. vivax* infection, and show potential for use as predictors of recent *P. vivax* infection.

**Conclusions:**

To achieve *P. vivax* elimination goals, novel diagnostics are required to aid in detection of hidden parasite reservoirs. PvRBP2b was previously shown to be the top candidate for single-antigen classification of recent *P. vivax* exposure and here, it is concluded that several alternative recombinant PvRBP2b fragments can achieve equal sensitivity and specificity at predicting recent *P. vivax* exposure.

**Supplementary Information:**

The online version contains supplementary material available at 10.1186/s12936-022-04085-x.

## Background

*Plasmodium vivax* is the world’s most widely distributed species of *Plasmodium* to infect humans causing malaria, and in near- and pre-elimination settings is proving more challenging to successfully eliminate [1]. In 2019, there were an estimated 229 million cases of malaria across all five of the World Health Organization (WHO) regions [2]. Cases of both *P. vivax* and *Plasmodium falciparum* are seen in all regions, however infection from *P. falciparum* is overwhelmingly the predominant cause of malaria (93% of all cases), driven particularly by infections throughout sub-Saharan Africa [2]. Despite this, as total malaria cases decrease, the proportion of infections that are attributable to *P. vivax* outside Africa is increasing and overall reductions in case rates are slower for *P. vivax *[3, 4].

*P. vivax* is a relapsing form of *Plasmodium*. During liver stage development, *P. vivax* matures towards the infective blood-stage by forming hepatic schizonts, but can also become developmentally arrested as a hypnozoite making an individual at risk of future relapsing episodes either weeks or months after the initial infection (when an individual is not treated with anti-hypnozoite drugs) [5, 6]. Most *P. vivax* blood-stage infections, which also produce transmissible gametocytes, have been shown to be the product of a relapsing hypnozoite as opposed to new transmission events of primary infection [7–9]. Current diagnostics to detect *Plasmodium* infection in programmatic settings rely on either the use of rapid diagnostic tests (detecting parasite antigens in the blood) or microscopy of Giemsa-stained blood-smears [2]. Both these methods will only detect a current blood-stage infection, hence missing all asymptomatic hypnozoite infections. These methods are also insensitive to the common low parasitaemic infections of *P. vivax *[3, 10]. In the context of *P. vivax* elimination, the overwhelming majority of infectious *P. vivax* episodes that are caused by relapsing hypnozoite infections reiterates the need to innovatively target this reservoir of disease [11]. The ability to identify individuals who are infected with hypnozoites would be revolutionary in *P. vivax* control, however the sparsity of infection throughout the liver and the inaccessibility of this organ makes this exceptionally challenging [12, 13].

Previous work has identified a novel panel of eight recombinant *P. vivax* antigens that can be used to detect antibody responses indicating exposure to *P. vivax* blood-stage infection within the last 9-months, with 80% sensitivity and 80% specificity [14]. These antigens were down-selected from a starting panel of more than 300 *P. vivax* proteins and validation studies of the eight selected *P. vivax* proteins were conducted in multiple geographic and epidemiological settings. Using this data, mathematical modelling demonstrated that with a *P. vivax* serological test and treat (PvSeroTAT) regimen, there is the potential to reduce *P. vivax* PCR prevalence by 59–69% in endemic areas [14] if anti-hypnozoite treatment with 8-aminoquinolines is provided to those identified as recently infected.

This in-development test requires further optimization and testing of different protein constructs to ensure sensitivity and specificity is maximized, potentially by reducing background cross-reactivity of the antigens in the assay. Previously, two different recombinant proteins of *P. vivax* reticulocyte binding protein 2b (PvRBP2b) were identified as the best single antigen predictors of recent *P. vivax* infection in a 9-month time frame [14]. Therefore, in the current study the primary objective was to test the performance of multiple fragments within the N-terminal and C-terminal domains of PvRBP2b expressed in either *Escherichia coli* or a Wheat Germ Cell Free (WGCF) expression system. In summary, several protein fragments were identified that detect antibody responses associated with recent infection to a similar or equal capability as the current top predictors.

## Methods

### Protein expression and magnetic bead coupling

**Fig. 1:**
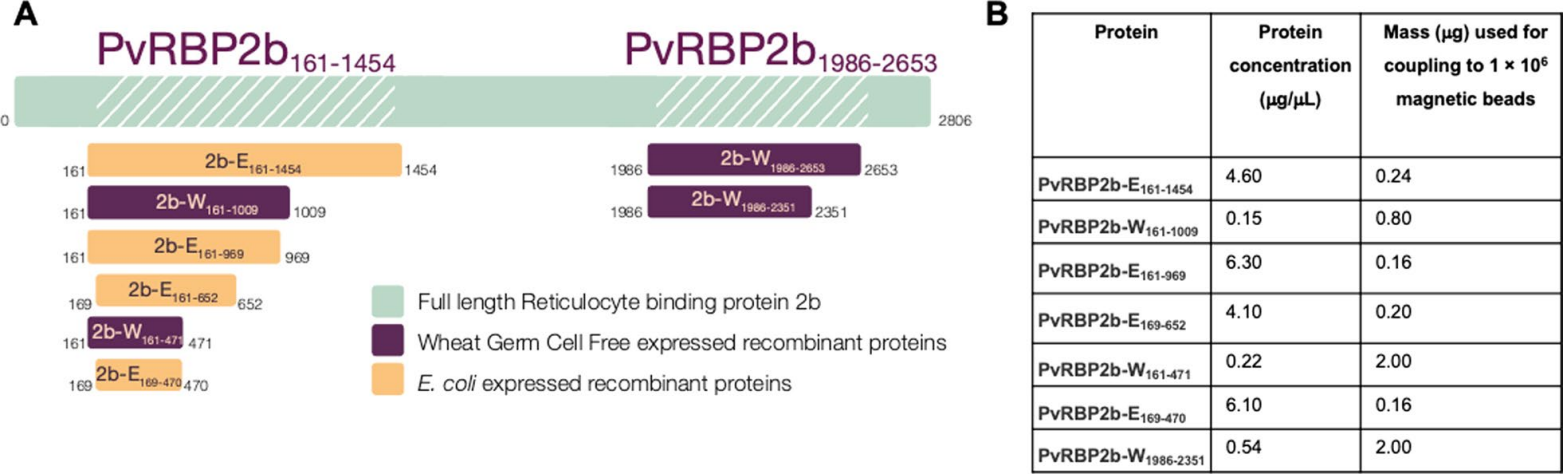
**A** Schematic of full-length PvRBP2b protein and regions encompassed by recombinant protein fragments included in this study (with amino acid positions denoted below) **B** Corresponding protein concentrations (µg/µL) and mass of protein (µg) used in bulk couplings (magnetic beads). The various amounts optimized here fall within the standard range described for other serological marker proteins using magnetic beads [18]

Recombinant proteins used in this study were expressed in either an *Escherichia coli *[15] or a WGCF expression system [14] (and denoted subsequently with E or W in protein names, respectively). Protein fragment size was chosen according to domain boundaries and homology to other RBP family proteins (notably PvRBP2a) [16]. Proteins denoted PvRBP2b-E_161–1454_ and PvRBP2b-W_1986–2653_ were top performing predictors of recent *P. vivax* infection in a previous study [14], and are N-terminal and C-terminal fragments, respectively (Fig. 1A). All subsequent smaller proteins were the focus of the current study in comparison to the previously identified top predictors (Fig. 1A). The *E. coli* proteins had previously been expressed as described [15], whilst the WGCF constructs denoted PvRBP2b-W_161–1009_, PvRBP2b-W_161–471_ and PvRBP2b-W_1986–2351_ were produced for the first time following standard methods for WGCF expression, and as previously described [14]. An exception was that PvRBP2b-W_161–471_ required addition of 0.05% Brij35 detergent during the translation and purification steps to prevent protein aggregation. Merozoite surface protein 1–19 (PvMSP1-19) was also used and was previously expressed in WGCF as described [14]. Purified recombinant protein was visualized via sodium dodecyl sulphate polyacrylamide gel electrophoresis (SDS-PAGE) (10% SDS) (Invitrogen NuPAGE, USA, catalogue number: NP0335BOX) and stained with SimplyBlue^™^ Safe Stain (Invitrogen, USA, catalogue number: LC606), Additional file 1: Fig. S1.

The recombinant proteins described were covalently coupled to magnetic microspheres (BioRad Laboratories, USA) in concentrations that were determined by a standard curve serial dilution to achieve a log-linear relationship. This allows plasma samples to be run at one dilution (1:100). Briefly, the carboxyl functional groups of the magnetic microspheres were activated with N-hydroxysulfosuccinimide (Sigma, USA) and N-(3-Dimethylaminopropyl)-N-ethylcarbodiimide (Sigma, USA) and then proteins were incubated with activated beads for either 2 h at room temperature or overnight at 4 °C, on continuous rotation. The microspheres were then washed and resuspended in phosphate buffered saline with 0.1% BSA, 0.02% Tween-20, 0.05% sodium azide. The magnetic microspheres are internally color-coded, thus allowing multiplexing in the final antibody assay. The amounts of protein coupled per bead region is detailed in Fig. 1B.

### Measurement of Immunoglobulin G (IgG) in plasma

IgG was measured in plasma samples according to the previously established multiplexed Luminex assay based on xMAP® technology [17], with minor adjustments made for use of magnetic microspheres with the MAGPIX® machine. Briefly, diluted plasma in a 1:100 concentration was incubated in singlicate with protein coupled beads for 30 min on a plate shaker. No technical replicates were used due to the repeatability of this assay [18, 19] as well as the large number of biological replicates. Following washing, a secondary antibody specific to the Fc region of total IgG (Jackson ImmunoResearch, USA, category number JI709116098) was incubated with the beads, detecting bound antibodies (1:100 dilution). The secondary antibody is conjugated to fluorescent phycoerythrin for quantification on the MAGPIX®. A median fluorescence intensity (MFI) is obtained for each bead region and each sample in the multiplexed assay. A hyper-immune positive control pool of individuals from Papua New Guinea (PNG) was used to standardize samples run between plates, Additional file 1: Fig. S2 and as previously described [17]. Briefly, a five-parameter logistic regression curve is fit to the serially diluted pool and MFI is converted to relative antibody units (RAU). Measurements for protein PvRBP2b-W_1986–2653_ proved challenging with the magnetic beads as they formed large coagulates. For this reason, data presented in this manuscript for PvRBP2b-W_1986–2653_ was previously obtained from the Luminex®-200 system using non-magnetic beads [14] and included for comparative reasons. A systematic comparison of the MAGPIX® and the Luminex®-200 (method used previously for quantification) has shown these two platforms to be comparable [18]. All RAU data generated is provided in Additional file 2.

### Plasma cohort samples

A 12-month longitudinal cohort of plasma samples from Ratchaburi and Kanchanaburi Provinces in Thailand collected in 2013/2014 was used for this study [20]. 809 patient plasma samples from the final visit of this study were assayed for total IgG to the selected recombinant proteins. An individual’s *P. falciparum* or *P. vivax* infection history during the twelve-month study was monitored by monthly qPCR, and epidemiological details from this study are published [20]. The antibody responses to the PvRBP2b protein fragments were additionally compared in a Thai Clinical cohort conducted in Tha Song Yang, Tak Province, Thailand, where 34 individuals completed a nine-month follow-up study following presentation with symptomatic *P. vivax *[21]. Plasma samples from the time of presentation with clinical *P. vivax* and one-week later were used.

Additionally, to monitor naïve and background reactivity to these recombinant *P. vivax* proteins, antibody levels were measured in 274 individuals with no known malarial infection history from Melbourne, Australia and Bangkok, Thailand. These were collected from the Australian Red Cross or the Volunteer Blood Donor Registry (WEHI) for the Australian samples, and from the Thai Red Cross in Bangkok for the Thai Samples.

### Statistical analyses

#### Correlations and significance testing

Antibody correlations were calculated using the non-parametric Spearman’s rho test and visualized using corrplot in R [22]. The non-parametric Mann–Whitney U test for significance was performed in Prism v7.0d (GraphPad Software, USA).

#### Classifying recent infection

Individuals were classified as having a recent infection if they had a PCR detectable *P. vivax* infection within the last nine-months of the study period (inclusive of current infections). Sensitivity and specificity were calculated at 10,000 intervals between maximum and minimum of detection limits and subsequent Receiver Operator Characteristic (ROC) curves demonstrate the sensitivity and specificity relationship of single antigen classification. Area under the curve (AUC) was calculated for each protein relative to its ROC curve. A linear discriminant analysis was also used to combine the RAU of an PvRBP2b protein fragment with another top performing protein, PvMSP1-19, and sensitivity and specificity were once again calculated. The classification algorithm was performed in R as previously described [14].

## Results

### **Immunogenicity of PvRBP2b protein fragments in individuals exposed to*P. vivax***

**Fig. 2:**
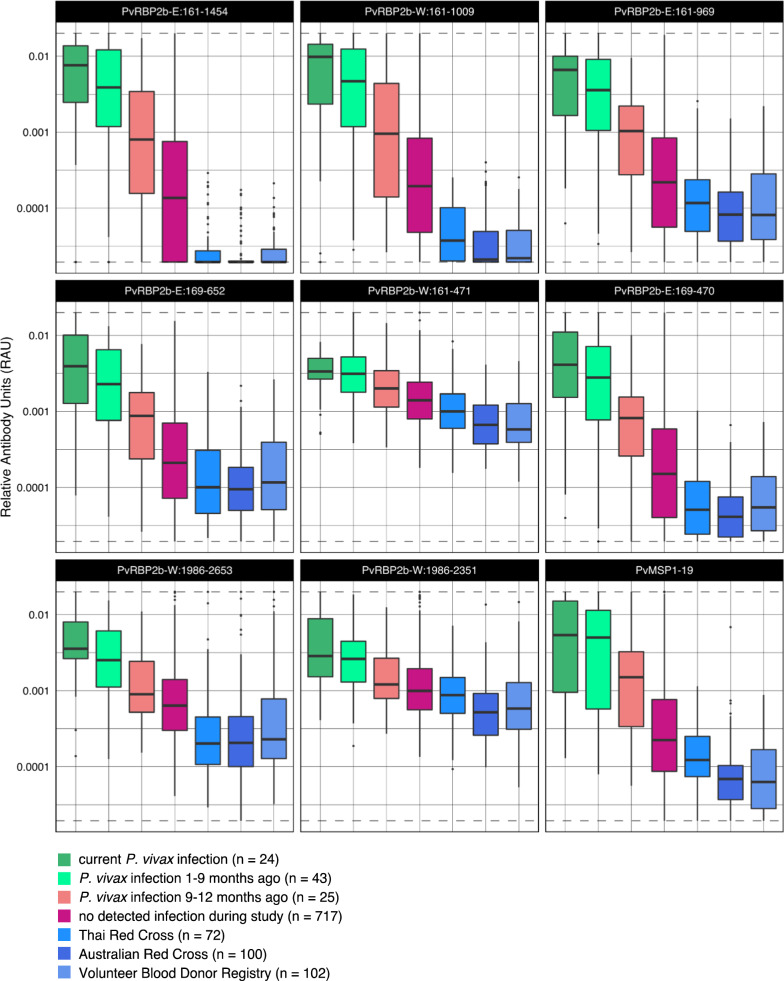
Measured IgG antibodies against various *P. vivax* RBP2b protein fragments and PvMSP1-19 in individuals from a low malaria-transmission region in Thailand, categorized by time since prior *P. vivax* infection. Boxplots denote median and interquartile range of antibody levels and whiskers indicate ±1.5 interquartile range (points outside of this range are shown individually), in Relative Antibody Units (RAU). IgG levels from three panels of malaria-naïve negative controls are also shown

Six smaller protein fragments of PvRBP2b-E_161–1454_ and PvRBP2b-W_1986–2653_ were used to measure antibody responses in a 12-month observational Thai cohort to assess the suitability for use in a serological intervention diagnostic tool and to see if the different protein fragments could result in improvements in classification accuracy. The first aim was to determine whether the smaller protein fragments were immunogenic in individuals with recent *P. vivax* infections (either currently or in the last nine months) compared to the non-exposed controls as well as individuals from the cohort with no detected exposure. Most of the smaller N-terminal protein fragments (PvRBP2b-W_161–1009_, PvRBP2b-E_169–470_, PvRBP2b-E_161–969_ and PvRBP2b-E_169–652_) show similar trends in declining antibody levels with increasing time since prior *P. vivax* exposure (Fig. 2). For all proteins, the highest median level of antibody response, measured in relative antibody units (RAU), was detected in those who were currently infected with *P. vivax* or had been infected within the last nine-months. Importantly, the lowest median level of antibody response for these N-terminal protein fragments (PvRBP2b-W_161–1009_, PvRBP2b-E_169–470_, PvRBP2b-E_161–969_, PvRBP2b-E_169–652_) was seen in individuals who had no known exposure to *P. vivax* (Fig. 2). This pattern was not observed against the two smaller protein fragments PvRBP2b-W_161–471_ and PvRBP2b-W_1986–2351_ of N-terminal and C-terminal ends of PvRBP2b, respectively, where there was very little difference in antibody responses detected between individuals with a recent infection and those with no known exposure to *P. vivax*, due to elevated levels in these non-malaria exposed controls (Fig. 2). There was also very little variation observed in antibody responses between any category of recent *P. vivax* infection (Fig. 2). The lowest background reactivity levels in the malaria-naïve control panels were observed for one of the original PvRBP2b constructs, PvRBP2b-E_161–1454_.

**Fig. 3:**
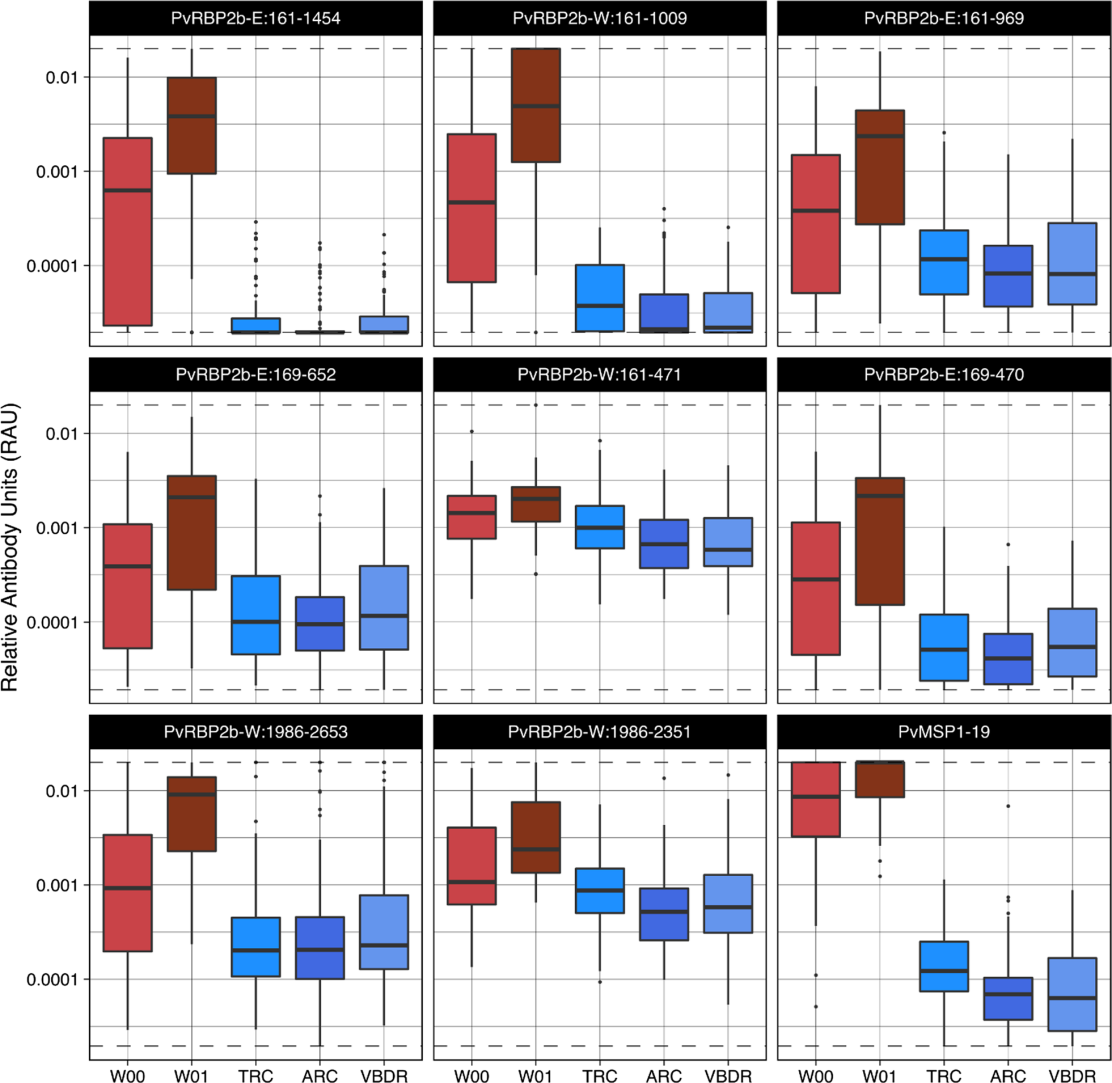
Measured IgG antibodies against various *P. vivax* RBP2b protein fragments following symptomatic *P. vivax* infections in Thailand. The antibodies in plasma of 34 individuals were measured at presentation of symptomatic *P. vivax* infection (W00) and one week later (W01) and compared to malaria naïve negative controls from the Volunteer Blood Donor Registry (VBDR), Australian Red Cross (ARC) and Thai Red Cross (TRC). Box plots show median Relative Antibody Units (RAU) and the interquartile range and whiskers indicate ±1.5 interquartile range (points outside of this range are shown individually). Statistical difference in RAU at W1 compared to each of the panels of negative controls were assessed using Mann−Whitney U test, all p < 0.0001 except for PvRBP2b-W_161–471_ when compared to the TRC panel

Most individuals with *P. vivax* in the Thai cohort were asymptomatic. Hence, total IgG responses to these protein fragments were further assessed in a symptomatic *P. vivax* cohort where samples were obtained at time of presentation to clinic and one week later (n = 34 individuals), capturing the initial boost of antibodies following infection. A significantly higher total IgG antibody response one-week post infection was observed compared to the negative controls for all proteins, (p < 0.0001 for all except PvRBP2b-W_161–471_ p < 0.001, Mann-Whitney U test) (Fig. 3). As per the data from the 12-month cohort of mainly asymptomatic individuals, there was less distinction in IgG levels between the symptomatic individuals and the negative controls for PvRBP2b-W_161–471_ and PvRBP2b-W_1986–2351_ compared to the other fragments.

### Correlation of total IgG antibody responses detected amongst recombinant PvRBP2b protein fragments

**Fig. 4:**
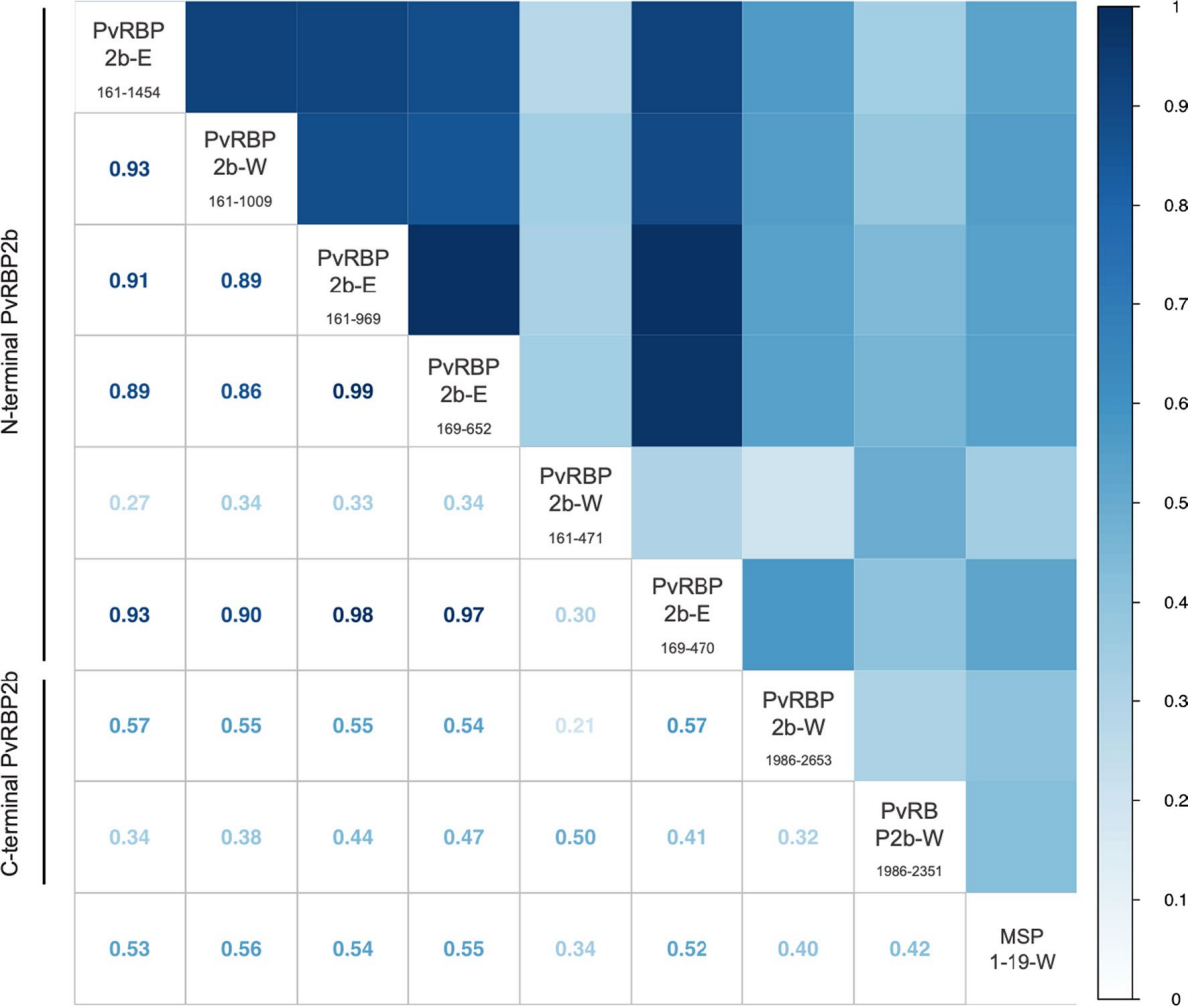
Correlation plot of anti-*P. vivax* RBP2b antibody responses against the various protein fragments as well as PvMSP1-19 for comparison. IgG antibody levels were measured at the last visit of the 12-month Thai observational cohort (n = 809), and correlation coefficients calculated using Spearman’s rho. All coefficients are significant, p < 0.0001

To investigate the association of antibody levels to different fragments, pair-wise correlations (non-parametric Spearman’s rho coefficient) were conducted between the antibody responses detected for each of the 809 individuals in the Thai cohort as shown in Fig. 4. All correlation coefficients between the PvRBP2b protein fragments were significantly and positively correlated but the strength of this correlation varied between 0.21 and 0.99. The most strongly correlated proteins were the N-terminal protein fragments, irrespective of expression system, except for protein fragment PvRBP2b-W_161–471_, a clear outlier; where all correlation coefficients between other fragments were less than 0.5. Additionally, the correlation coefficients between N-terminal fragments (not including PvRBP2b-W_161–471_) and C-terminal fragment top performer PvRBP2b-W_1986–2653_ ranged between 0.54 and 0.57. The correlation between the two smallest fragments, PvRBP2b-W_161–471_ and PvRBP2b-E_169–470_, which encompass almost the same amino acid sequence (difference in 9 amino acids), was a very weak positive correlation with a Spearman’s rho value of 0.3. These two proteins were expressed in two different expression systems, the WGCF and *E. coli*, respectively. In contrast, the correlation coefficients of the other N-terminal fragments ranged between 0.86 and 0.99 (Fig. 4). N-terminal fragments (not including PvRBP2b-W_161–471_) and PvMSP1-19 were less well-correlated with correlation coefficients ranging from 0.52 to 0.56 (Fig. 4).

### **Classifying individuals with a recent*P. vivax*infection**

**Fig. 5:**
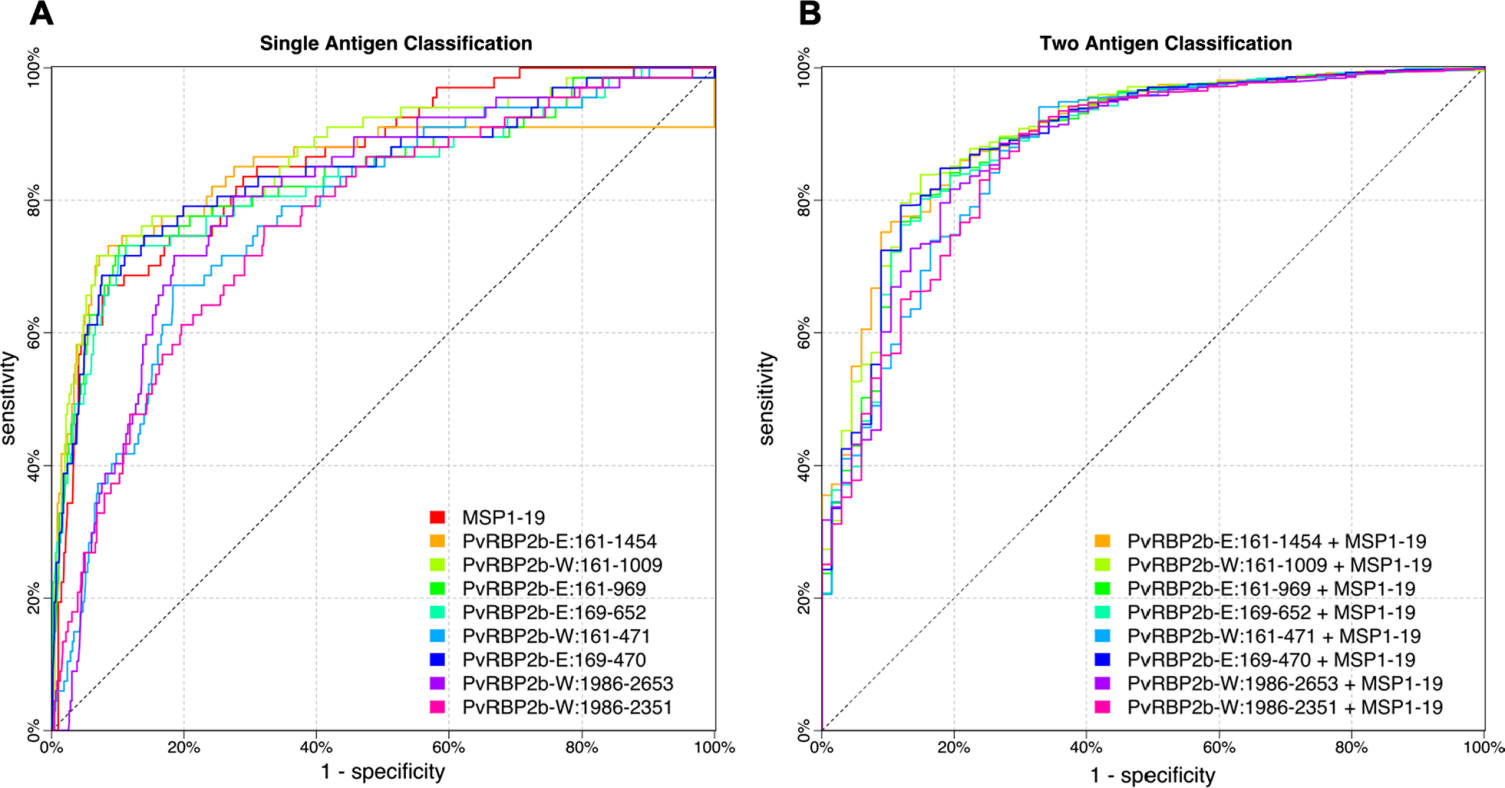
Receiver operator characteristic curves. **A** Sensitivity and specificity of classification using total IgG antibodies against only one *P. vivax* recombinant protein to predict recent infection and **B** Sensitivity and specificity of classification of recent infection using one of each of the PvRBP2b fragments and another top predictor of recent infection, PvMSP1-19. The receiver operator characteristic curves present the ability of the algorithm to correctly classify individuals as recently infected with *P. vivax* in the prior 9-month period, or not, based on PCR-detected infections

The next objective was to assess the ability of total IgG antibodies against each recombinant PvRBP2b protein fragment to classify an individual within the cohort as being recently or non-recently exposed to *P. vivax*. To do this, a classification algorithm that assessed sensitivity and specificity was used at a range of antibody detection levels for each single antigen. Recent *P. vivax* infection was defined as occurring within the prior nine-month period, as per previous analyses [14]. PvRBP2b-E_161–1454_ was previously identified as the top predictor of recent infection and classification in this study was compared relative to PvRBP2b-E_161–1454_’s classification capability. The best performing fragments were PvRBP2b-W_161−1009_, PvRBP2b-E_169–652_, PvRBP2b-E_169–470_, PvRBP2b-E_161–969_ as they had very similar classification capacities (Fig. 5 A), as shown by the AUC values calculated ranging between 0.83 and 0.87, in this cohort (Table 1, compared to 0.86 for PvRBP2b-E_161–1454_). The second-best performing protein in previous analyses was the merozoite surface protein (PvMSP1-19) [14]. As this protein is already of very short length (108 aa), no further optimization of the construct size was deemed necessary. To assess combinatorial prediction of two antigens to classify recently exposed individuals, a linear discriminant analysis was used to classify and therefore determine sensitivity and specificity. The addition of PvMSP1-19 led to improved classification performance when combined with all fragments of PvRBP2b, bringing all AUC values to a small range of 0.87 to 0.90 (Fig. 5B; Table 1). The most notable increases were when PvMSP1-19 was combined with the PvRBP2b fragments that had the lowest classification on their own; particularly PvRBP2b-W_161–471_ and PvRBP2b-W_1986–2351_ (Fig. 5B).

**Table 1:** Area under the curve values for single antigen classification and for two antigen classification (with PvMSP1-19)

	PvRBP2b-E_161−1454_	PvRBP2b-W_161–1009_	PvRBP2b-E_161–969_	PvRBP2b-W_161–471_	PvRBP2b-E_169–470_	PvRBP2b-E_169–652_	PvRBP2b-W_1986–2653_	PvRBP2b-W_1986–2351_	PvMSP1-19
Single antigen	0.86	0.87	0.84	0.78	0.85	0.83	0.80	0.77	0.86
PvRBP2b fragment + PvMSP1-19	0.90	0.90	0.89	0.87	0.89	0.89	0.88	0.87	NA

## Discussion

The use of a serodiagnostic test to identify and target individuals for treatment, who are likely to have had recent exposure to *P. vivax*, is a novel and potentially transformative approach to achieving *P. vivax* elimination in low-transmission settings [23], like that presented in this study in Western Thailand. PvRBP2b is a parasite protein essential for reticulocyte invasion by the merozoite during blood-stage infection [15], and antibodies against two recombinant PvRBP2b protein fragments have previously been identified from a large screen to be the best single-antigen predictors of a *P. vivax* infection within the last 9 months [14]. Here the primary objective was to validate these findings and explore the suitability of smaller, potentially more specific protein fragments to classify recent *P. vivax* infections. It was hypothesized that smaller protein fragments would improve specificity of the classification by reducing background reactivity to portions of the proteins that were non-immunogenic. This hypothesis was based on earlier data where a significant negative correlation was observed between background antibody reactivity levels in malaria-naïve individuals and the AUC values from the classification algorithm [14]. In the current study, significant improvements of classification were not observed beyond the two original PvRBP2b proteins. This is likely due to the strong correlation between antibody levels measured to most PvRBP2b fragments assessed and no reduction in background reactivity in the negative controls using the smaller fragments compared to the original constructs.

In this study, it was observed that despite almost identical amino acid sequences (differing by 9 amino acids), PvRBP2b-E_169–470_ outperformed PvRBP2b-W_161–471_ in terms of classifying recent exposure to *P. vivax*. A potential reason may be protein folding, suggesting the antibodies targeted to PvRBP2b-E_169–470_ could be to conformational epitopes as opposed to linear epitopes. The two constructs were expressed using different expression systems (PvRBP2b-W_161–471_ WGCF versus PvRBP2b-E_169–470_ *E. coli*), however the other pair of fragments expressed in different expression systems performed similarly to each other and were well correlated (PvRBP2b-W_161–1009_ WGCF vs. PvRBP2b-E_161–969_ *E. coli*). Furthermore, PvRBP2b is thought to be under balancing selection, particularly between amino acid positions 169–470 and where there is increased nucleotide diversity amongst *P. vivax* genomes [15]. It may therefore be worthwhile to systematically assess the impact of different polymorphisms on the subsequent antibody response to PvRBP2b. However, prior data has indicated that PvRBP2b performs well as a serological exposure marker in Thailand, Brazil and the Solomon Islands; three regions of differing *P. vivax* genetic diversity and, therefore, it is not expected that these polymorphisms would substantially impact the performance of this marker [14]. Ultimately, the most likely hypothesis for why PvRBP2b-W_161–471_, and also PvRBP2b-W_1986–2351_, performed poorly at classifying recent *P. vivax* infections is the high background reactivity observed in the malaria-naïve negative control panels. This could be due to the high amount of protein required for coupling to see a clear signal in the positive control pool for these proteins. Only one dilution of plasma (1:100) was tested, this is a possible limitation and further dilution series could be tested to see whether a better signal to noise ratio could be obtained. The poor performance of PvRBP2b-W_161–471_ and PvRBP2b-W_1986–2351_ is also highlighted by the lack of correlation with the other PvRBP2b fragments, with correlation coefficients lower than with the unrelated protein PvMSP1-19.

The findings from this study further support the original finding that PvRBP2b-E_161–1454_ and PvRBP2b-W_1986–2653_ (non-overlapping N and C terminal constructs of PvRBP2b) induce correlated IgG antibodies that both can accurately classify individuals as recently exposed to *P. vivax *[14]. This suggests that in the context of designing proteins for use as serological markers of exposure, in contrast to designing proteins for vaccines, multiple constructs and fragments could be used if antibodies are co-acquired to multiple epitopes. The choice of final protein construct (from those that had similarly high performance) will depend instead on other characteristics, such as stability, yield and/or purity, for example. Furthermore, it is shown that protein fragments non-exclusively expressed in the same expression system can work as well as each other and this provides important practical advantages in terms of the development of a scalable serological marker intervention tool where protein choice and expression system can play an important role. Future plans include a systematic comparison of multiple protein expression systems for producing the top *P. vivax* serological marker proteins in the next phase of this work.

## Conclusions

In conclusion, there was no smaller fragment of PvRBP2b that out-rightly improved classification performance, however it was found that multiple PvRBP2b protein fragments can be used to give near-equal classification performance. Importantly, background reactivity was already low to PvRBP2b-E_161−1454_ compared to other proteins identified previously to be the best predictors of recent infection [14]; therefore, there may still be benefit in fragment optimization for some of the other longer protein constructs within the top 8, such as PvMSP3 (construct length 828 aa [14]).

## Supplementary information

**Additional file 1: Figure S1. **SDS-PAGE (10%) visualisation of purified recombinant proteins used for magnetic bead coupling. **Figure S2. **Standard curve serial dilutions for plates used in this study of protein-magnetic bead couplings with the hyper-immune positive control pool of individuals from Papua New Guinea.

**Additional file 2.** Supplementary data supporting results. **Sheet 1.** Relative Antibody Units (RAU) for individuals in the 12-month longitudinal cohort conducted in Thailand. **Sheet 2.** Relative Antibody Units (RAU) for individuals in the symptomatic cohort at time of infection and one-week post infection. **Sheet 3.** Relative Antibody Units (RAU) for individuals in the three negative control cohorts.

## Data Availability

All data generated and analysed is included in the additional files.

## Abbreviations

PvRBP2b: *Plasmodium vivax* Reticulocyte binding protein 2b
IgG: Immunoglobulin G
PCR: Polymerase chain reaction
WGCF: Wheat germ cell free
PvMSP1-19: *Plasmodium vivax* merozoite surface protein 1–19
BSA: Bovine serum albumin
MFI: Median fluorescence intensity
RAU: R elative antibody units
ROC: Receiver operator characteristic
AUC: Area under the curve
aa: Amino acid
