# Supplementary material for: Comparison of total immunoglobulin G antibody responses to different protein fragments of Plasmodium vivax Reticulocyte binding protein 2b
Source: Malar J. 2022 Mar 4;21:71. doi: 10.1186/s12936-022-04085-x (PMC8896302; doi:10.1186/s12936-022-04085-x)
Supplement: Supplementary file 1 — Additional file 1: Figure S1. SDS-PAGE (10%) visualisation of purified recombinant proteins used for magnetic bead coupling. Figure S2. Standard curve serial dilutions for plates used in this study of protein-magnetic bead couplings with the hyper-immune positive control pool of individuals from Papua New Guinea. [file 12936_2022_4085_MOESM1_ESM.docx]

Supplementary Figures


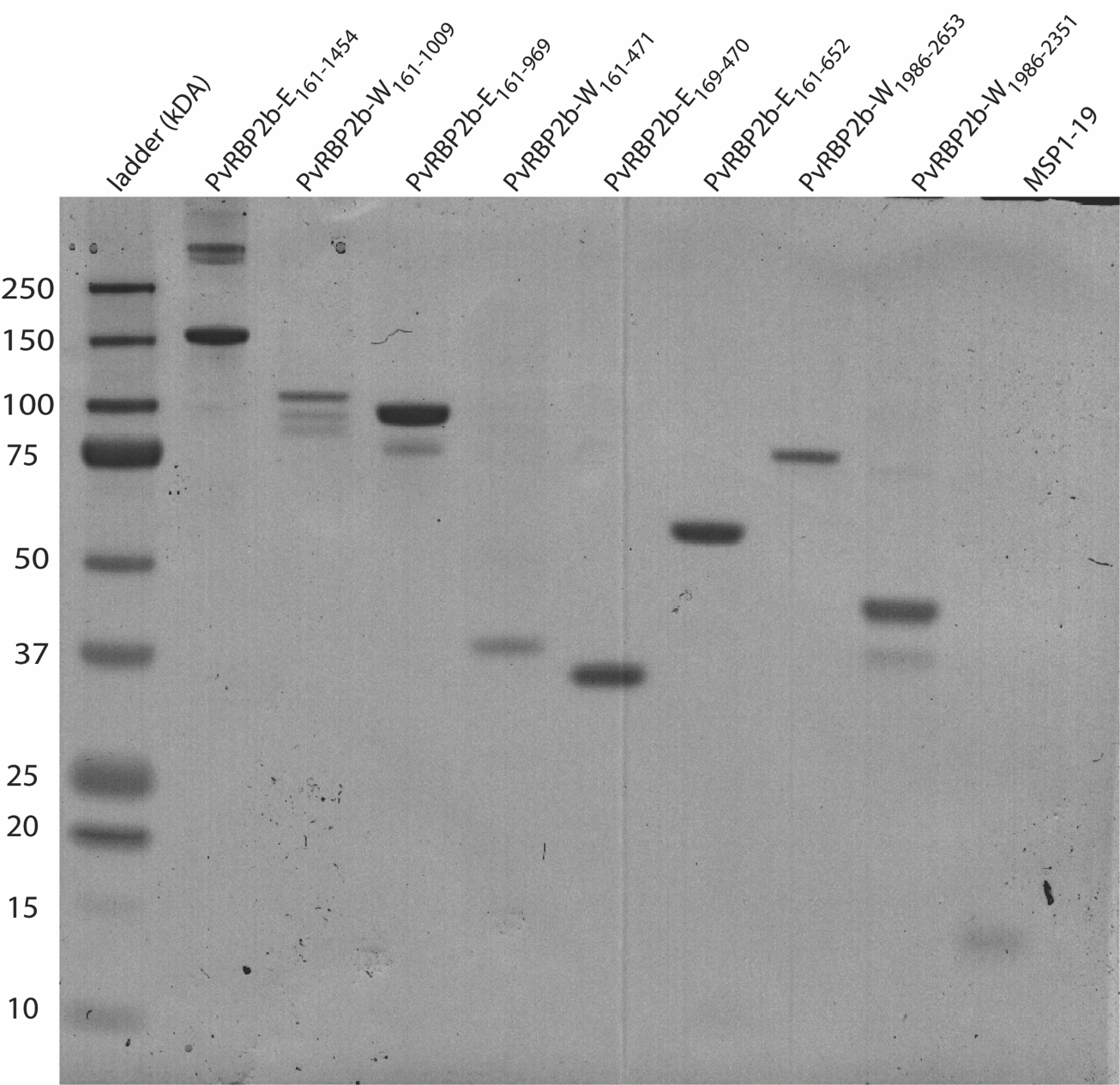


**Supplementary Figure 1.** SDS-PAGE (10%) visualisation of purified recombinant proteins used for magnetic bead coupling.


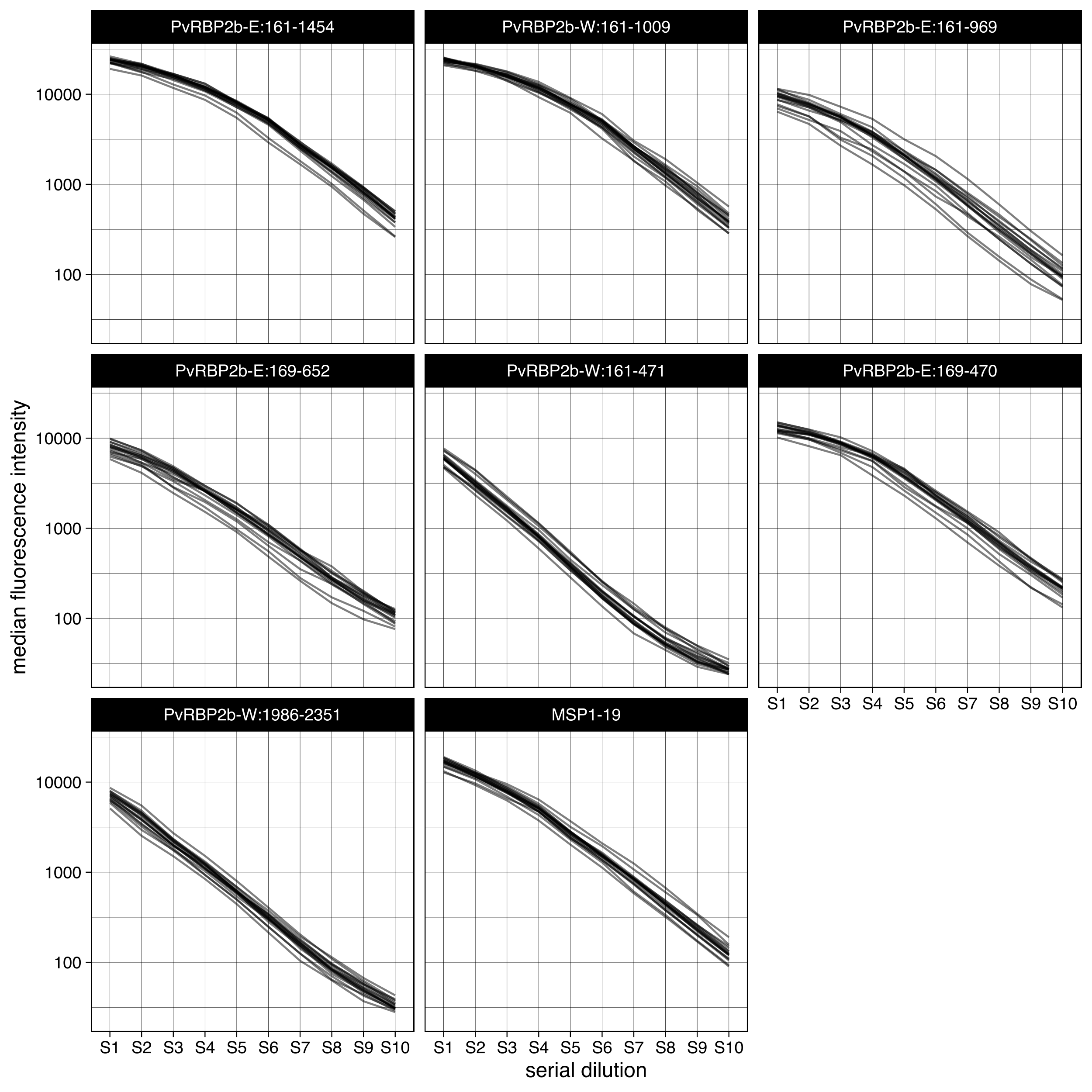


**Supplementary Figure 2.** Standard curve serial dilutions for plates used in this study of protein-magnetic bead couplings with the hyper-immune positive control pool of individuals from Papua New Guinea.
